# Ice nucleation active particles are efficiently removed by precipitating clouds

**DOI:** 10.1038/srep16433

**Published:** 2015-11-10

**Authors:** Emiliano Stopelli, Franz Conen, Cindy E. Morris, Erik Herrmann, Nicolas Bukowiecki, Christine Alewell

**Affiliations:** 1 Environmental Geosciences, University of Basel, CH-4056 Basel, Switzerland; 2 INRA, UR0407 Pathologie Végétale, F-84143 Montfavet cedex, France; 3 Laboratory of Atmospheric Chemistry, Paul Scherrer Institut, CH-5232 Villigen PSI, Switzerland

## Abstract

Ice nucleation in cold clouds is a decisive step in the formation of rain and snow. Observations and modelling suggest that variations in the concentrations of ice nucleating particles (INPs) affect timing, location and amount of precipitation. A quantitative description of the abundance and variability of INPs is crucial to assess and predict their influence on precipitation. Here we used the hydrological indicator *δ*^18^O to derive the fraction of water vapour lost from precipitating clouds and correlated it with the abundance of INPs in freshly fallen snow. Results show that the number of INPs active at temperatures ≥ −10 °C (INPs_−10_) halves for every 10% of vapour lost through precipitation. Particles of similar size (>0.5 μm) halve in number for only every 20% of vapour lost, suggesting effective microphysical processing of INPs during precipitation. We show that INPs active at moderate supercooling are rapidly depleted by precipitating clouds, limiting their impact on subsequent rainfall development in time and space.

Ice formation in clouds contributes to the development of precipitation at mid-latitutdes^1^,^2^,^3^,^4^. Ice nucleating particles (INPs) of biological origin can be effective in promoting ice nucleation at temperatures around −10 °C or warmer^5^,^6^,^7^, whereas at colder temperatures inorganic substances are likely to be responsible for an increasing fraction of ice particles formed in the atmosphere^8^. Here we focus on the cumulative number of INPs active at temperatures warmer than −10 °C (INPs_−10_), the range where the activity of INPs of biological origin seems to be dominant. Such INPs include certain bacteria, fungal spores and pollen, but a large fraction of INPs from biological sources in the atmosphere may also be composed of ice nucleation active macromolecules associated with mineral and soil particles^9^,^10^. Because of usually very small number concentrations in the atmosphere, the potential role of such particles in conditioning precipitation is still contentious^11^,^12^.

Elevated concentrations of INPs associated with dust from desert storms on other continents and with far away and regionally emitted INPs were recently found to contribute to precipitation over the Western USA^4^ and the Amazon basin^13^ respectively. Overall, it is likely that there is a coincidence in time and space of the concentration of INPs and the intensity of precipitation events^14^, raising the general question of where and when cloud glaciation and subsequent precipitation are limited or facilitated by INPs. To address this question, it is crucial to understand the major factors driving the variation of atmospheric concentrations of INPs, which have been observed to range over several orders of magnitude^3^,^15^.

Feedbacks between human activities and climate modifications could be, or become, partly influenced by INPs. In fact, intensifying land use and climatic change are likely to increase future emissions of INPs associated with wind-blown soil dust^16^. Changes in vegetation cover, crop type and management may also affect emissions of ice nucleating particles from vegetation^12^. In this study we intend to quantify the relation between the fraction of water lost from air masses and the residual concentrations of INPs_−10_. This could help to assess the range of influence that a change in the source strength of INPs in a particular region may have on precipitation downwind.

## Results

### General overview

The concentration of INPs in an air mass is principally a function of (a) the accumulation of INPs from sea and land surfaces that the air mass has contacted, (b) the degree of mixing with other air masses, richer or poorer in INPs, and (c) the cumulative loss of INPs, most importantly by wet deposition processes, across its trajectory. Here we focus on the proportion of variation in the abundance of INPs that the last factor (c) might explain when (a) and (b) are presumed to be constant. Presuming (a) to be constant, we assume a temporally steady and spatially homogenous cumulative mix of INPs from several sources. Our presumption of (b) to be constant does not account for possible cumulative enhancement of INPs by falling and evaporating hydrometeors.

Observations and direct measurements relate to the conditions at the High Altitude Research Station Jungfraujoch (7° 59′ 06” E, 46° 32′ 51” N, 3580 m a.s.l.).

**Figure 1:**
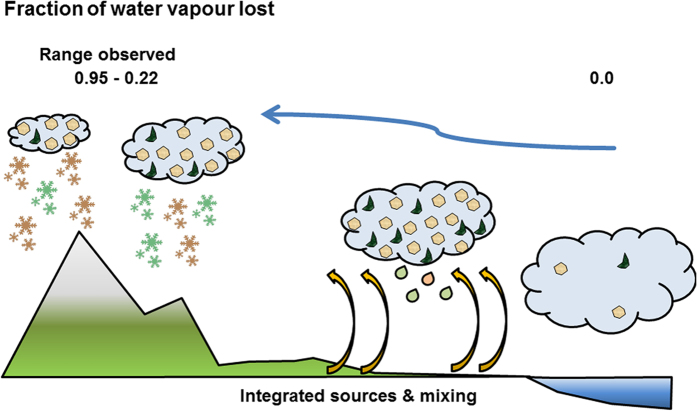
Relationship between the fraction of water vapour lost from a precipitating cloud (derived from stable isotope ratios in snow (*δ*^18^O)) and ice nucleating particles (INPs, measured in snow). As the cloud precipitates, the progressive loss of water vapour (from right to left) is accompanied by a loss of INPs which have been uplifted from the sea and land surfaces (yellow arrows). INPs of biological origin (green half-moons) are activated at more moderate supercooling, hence typically earlier than inorganic INPs (brown hexagons). The values 0.22 and 0.95 correspond to the minimum and maximum fractions of water vapour lost that we observed at Jungfraujoch (drawn by E. Stopelli).

Water precipitating at Jungfraujoch generally originates from evaporation from either the North Atlantic or the Mediterranean Sea, depending on trajectories of air masses^17^. Upon its approach over land, moist air picks up additional dust and biogenic particles from various sources. Through precipitation it loses varying proportions of water and particles before arriving at Jungfraujoch, which is on the highest mountain ridge between the Mediterranean and the Atlantic water source regions. Isotopic fractionation leads to a preferential condensation and loss of heavier isotopes (^18^O and ^2^H) compared to the lighter ones (^16^O and ^1^H; for sake of brevity we will refer hereafter only to oxygen). This results in increasingly smaller values of the ratio ^18^O/^16^O, expressed as *δ*^18^O, both in cloud water and rain or snow during the progressive development of precipitation^18^,^19^,^20^,^21^. Although the isotopic signal is sensitive to the integrated amount of precipitation deposited from an air mass, it provides no details about the specific conditions that have triggered a precipitation event (e.g. temperature) or whether the integrated precipitation was lost in one or in several events. Not only *δ*^18^O, but also the number of INPs in precipitation is influenced by the cumulative history of water loss from the air mass, since INPs active at the warmest supercooling are the first to be activated as a cloud progressively cools down, and are potentially removed with precipitation. Therefore, the larger the fraction of water that has precipitated, the greater the chance that INPs active at moderate supercooling have been removed, hence the smaller the fraction of such INPs among other particles in later precipitation (Fig. 1).

### INPs get rapidly lost from a precipitating cloud

**Figure 2:**
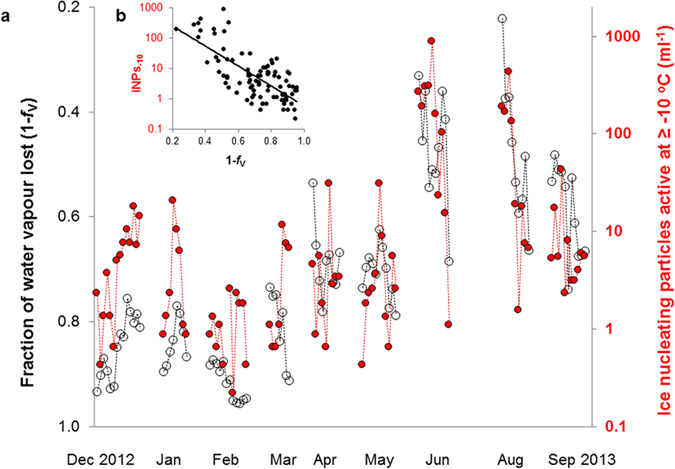
(**a**) Covariation over time of concentrations of INPs_−10_ in snow (red, log scale) and the estimated fraction of water vapour lost from an air mass prior to its arrival at the Jungfraujoch observatory (black). Time proceeds from left to right, intervals are not to scale. Each symbol signifies a snow sample with a median sampling duration of 2 hours. Each campaign (symbols connected by dotted lines, one campaign per month) lasted from 3 to 5 subsequent days. A total of 91 samples were collected between December 2012 and September 2013 (exception of July 2013 due to the lack of considerable precipitation events); (**b**) Relationship between INPs_−10_ and 1-*f*_V_; the function of the fitted exponential curve is reported in equation (1).

Over a 10-month period (December 2012 to September 2013) we sampled snow within precipitating air masses that had lost between 22 and 95% of their initial water content before arriving at the observatory (Fig. 2). The decision to initiate a sampling campaign depended on weather forecasts that predicted snowfall for at least two full days in a row, to assure that we could collect multiple samples within the same campaign. A total of 304 mm were collected, reaching approximately 20% of the total amount of precipitation fallen in the same period of 10 months at the closest station recording precipitation (1640 mm, Kleine Scheidegg, 2060 m a.s.l., 4.6 km North from Jungfraujoch). A trend of INPs_−10_ · ml^−1^ of snow with minimum values in winter and maximum values in summer appears from the data collected (comparison among months, Kruskall-Wallis p < 0.001). Nevertheless, similarly large variations of INPs_−10_ are apparent even among samples collected within a single sampling campaign, as for instance in June and August 2013.

The predominant factor with a similarly marked variability that correlates with the abundance of INPs_−10_ observed in precipitation, is the fraction of residual water vapour in clouds (Fig. 2).

The abundance of INPs_−10_ in snow declines exponentially with increasing proportions of water lost before arrival of an air mass at Jungfraujoch (Fig. 2b), and is best described by equation (1):


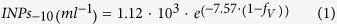



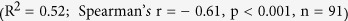


We can derive from this empirical equation, fitted to our data, an estimate for the largest number of INPs_−10_ to expect in 1 ml of precipitation at Jungfraujoch. If the very first precipitation from an air mass is just about triggered at the observatory (1–*f*_V_ = 0) we would expect it to contain around 10^3^ INPs_−10_ · ml^−1^. This number is probably a consequence of the strength of the sources of INPs influencing Jungfraujoch. Nevertheless, we can presume the exponential relationship to hold also in other places because of a generally geometric behaviour observed in precipitating particles^22^. Physical processes during the course of precipitation define the factor −7.57 in the exponent, which might have a similar value also at other locations where the same physical processes are at work. It suggests that the concentration of INPs_−10_ halves with about every 10% of moisture lost from a precipitating air mass (e.g. moving 1–*f*_V_ = 0.5 to 1–*f*_V_ = 0.6 results in: e^(−7.57 ⋅ 0.6)^/e^(−7.57 ⋅ 0.5)^ = 0.47).

### Selective removal of INPs

**Figure 3:**
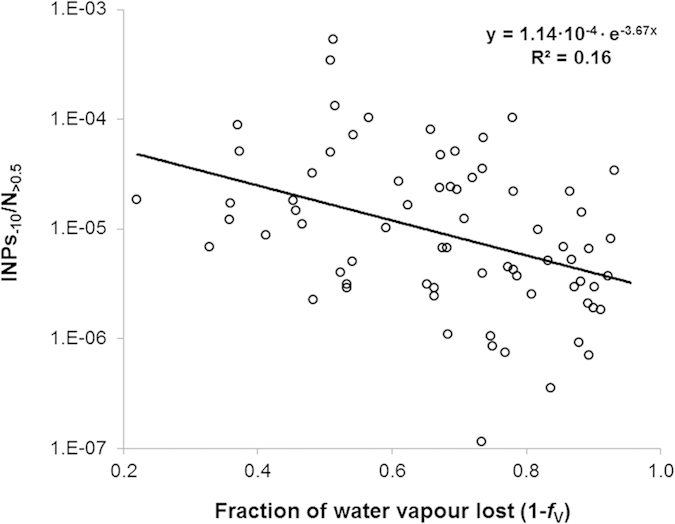
Ratio of INPs_−10_ to the total number of particles >0.5 μm (N_>0.5_, on log scale) as a function of the fraction of water vapour lost from the air mass prior to arrival at the observatory (n = 71, Spearman’s r = −0.42, p < 0.001).

The question remains whether there is experimental evidence for an INP being more likely to be deposited from an air mass than a particle of similar size that is not ice nucleation active. INPs should, in principle, be the starting point for snowflakes precipitating from a cloud. The average activation diameter for cloud condensation nuclei at Jungfraujoch is about 0.1 μm and most INPs are probably larger than 0.5 μm^23^,^24^. Relating concentrations of INPs_−10_ in precipitating snow to all particles larger than 0.5 μm (N_> 0.5_) in the same air volume reveals a significant negative trend in the ratio of INPs_−10_ to N_ > 0.5_ with an increasing fraction of vapour lost, despite the large scatter of data (Fig. 3).

If the ratio of INPs_−10_ to N_ > 0.5_ were independent from the fraction of water vapour lost, we could say that both kinds of particles are removed with equal efficiency from a precipitating cloud. This is clearly not the case. The function fitted to our data (Fig. 3) suggests that the ratio of INPs_−10_ to particles of similar size N_ > 0.5_ is reduced to 0.69 times of what it was before with every 10% of initial water vapour lost from a precipitating cloud (e.g.: e^(−3.67·0.6)^/e^(−3.67·0.5)^ = 0.69). With the same 10% loss of vapour, the absolute number of INPs_−10_ almost halves (0.47, equation (1)) and the absolute number of N_>0.5_ is consequently reduced to 0.68 times of what it was before (reduction of INPs_−10_ in absolute terms (factor 0.47) divided by the change in the ratio INPs_−10_/N_>0.5_ (factor 0.69) = 0.68). Hence, to halve the number of N_>0.5_ (0.68 × 0.68 = 0.46, approximately the half) requires about 20% moisture loss, almost twice the amount of water vapour lost to what is necessary to halve the number of INPs_−10_, suggesting active microphysical processing of particles^25^. This selective loss of INPs is highly significant, but explains only about one sixth of the total variation in the ratio of INPs_−10_ to N_>0.5_. The remaining variation might be due to source-related factors and could reflect temporal and spatial differences in INPs_−10_, N_>0.5_ and in the proportion of INPs among particles N_>0.5_ emitted to the atmosphere before precipitation. Part of the scattering of INPs_−10_ and N_>0.5_ data may be also due to differences in the dimensions of INPs and total particles in each sample. In fact, not only nucleation but also impaction scavenging of aerosols can contribute to the simultaneous removal of particles, with an efficiency largely depending on the size of aerosols and precipitation intensity^26^,^27^,^28^.

## Discussion

Land use and climate change alter the distribution, the quality and the size of soil and vegetation cover in a landscape, and with it the strength and distribution of sources of different INPs^12^,^16^,^29^. As we illustrate here, the ratio of stable water isotopes in precipitation can be used in novel way to characterize the history of air masses in terms of residual abundance of INPs. Despite simplifying assumptions, our approach explains more than 50% of the large variation of INPs_−10_ observed in snow both within short sampling campaigns and over the year. Much of the unexplained variation is probably due to variations in the initial concentration of INPs before precipitation, which depends both on the source strength of INPs and on the degree of the mixing of air masses with different initial concentrations of INPs (e.g. from different altitudes or regions). Source strength in the lowland north of Jungfraujoch is scattered over two orders of magnitude during most of the year, but does not seem to change with season^30^. In the same study the seasonal variation observed on Jungfraujoch seemed to be driven by microphysical processing of INPs through activation and deposition from approaching air masses. The same process may explain much of the observed temporal variation of INPs in this study (Fig. 2). It is in fact supported by the finding that INPs_−10_ are deposited more efficiently than other particles of similar size (Fig. 3).

Carrying out similar measurements on stable water isotopes and on INPs also at other stations will firstly lead to the constant improvement and refinement of our calculations and, secondly, it will shed new light on the evolution of concentrations of INPs before and during precipitation events over the trajectories of air masses.

This will provide an important contribution for mapping the probabilities of the abundance of INPs and their exchanges across regions, in particular the estimation of how far from a source and along a specific trajectory INPs might have an impact. Furthermore, the fact that INPs active at moderate supercooling get rapidly lost through precipitation adds a significant constraint to the role of such INPs on the development of cloud processes in time and space, fostering a deeper understanding of the effects of different land use strategies on rainfall distributions. Therefore, predictive modelling of precipitation patterns, and eventually of water supply and climate change could be improved through enhanced precision about where and when INPs are incorporated into air masses and subsequently parachute with rain and snow along their trajectory over landscapes.

## Methods

### Sample collection

During sampling events the Research Station Jungfraujoch was always inside clouds and the temperature of the air at the Station was ranging from −27.3 °C to 0.4 °C. Snow samples were collected with a teflon-coated tin (0.1 m^2^, 8 cm deep) carefully rinsed ethanol and sterile Milli-Q water. Sampling duration lasted from 1.5 to 8 hours (median time = 2 hours). Snow was melted at around 16 °C and analysed within less than 4 hours after its collection. The cumulative number of INPs was determined between −2 °C and −12 °C in immersion freezing mode, using an automated drop freeze apparatus^31^ loaded with 52 tubes containing 100 μl of sample each. In our analyses we concentrated on the warmest temperature at which all samples had a detectable number of INPs, which was −10 °C. The smallest number concentration of INPs that can be detected with this configuration is 0.21 INPs · ml^−1^. Blanks were periodically prepared by sprinkling Milli-Q water into the tin and analysed with the same material and method as the snow samples, at 200 μl per tube to obtain more conservative results. Blank values for INPs active at −10 °C were on average 0.11 INPs · ml^−1^, with only 7 blanks showing some freezing activity on a total of 39 blanks analysed.

### *δ* ^18^O analysis and modelling of *f*_V_ values

Aliquots of snow (equivalent to about 5 ml of water) were immediately loaded from the sampling tin into 15 ml sealed polypropylene Falcon tubes and stored at 4 °C until analysis with a tunable, off-axis integrated-cavity laser spectrometer (DLT−100, Los Gatos Research, Inc. (LGR), Mountain View, California). Standards used for calibration were provided by LGR and all results presented here were related to the standard VSMOW. The local meteoric water line obtained from the whole set of yearly data fits well with the equation associated to the global meteoric water line (*δ*^2^H = 7.7 *δ*^18^O + 10.6; R^2^ = 0.98). This indicates the absence of significant disequilibrium conditions at Jungfraujoch compared to the global behaviour of precipitations. The remaining water vapour fraction (*f*_V_) was calculated from *δ*^18^O‰ values measured in snow (*δ*_L_) following the method described in Rowley 2001^19^.

The evolution of *δ*^18^O in vapour (*δ*_V_) can be described by a Rayleigh-type fractionation model^21^,^32^:


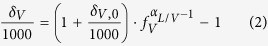


In our calculations, the fractionation factor from liquid to vapour *α*_L/V_ along the trajectory of the cloud was assumed constant during the entire path of a precipitating cloud and proportional to the average value between the temperature of the air at Jungfraujoch and the estimated temperature at the sea surface from where the air mass originated. The dependence of *α*_L/V_ from absolute temperature (*T*) was calculated according to Majoube 1971^33^:


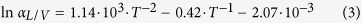


The isotopic ratio of the vapour at Jungfraujoch (*δ*_V_) was calculated from the isotopic ratio of snow (*δ*_L_) and the fractionation factor liquid-vapour at the temperature of the air recorded at the station:


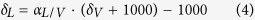


Seawater was considered the principal and constant source of moisture in calculating the isotopic ratio of the initial water vapour (*δ*_V,0_):


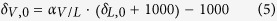


with the isotopic *δ*^18^O ratio of seawater (*δ*_L,0_) homogeneously equal to 0‰, since it coincides with the standard reference for water stable isotopes measurements and the fractionation factor between seawater and vapour *α*_V/L_, equal to 1/*α*_L/V_. Over the year, the station is affected by intermittent influence of the boundary layer^34^, with air masses coming from different geographical regions and its location in a saddle allows air systems to be channelled along two main directions, mainly North-West and South-East^35^. Vapour source regions were derived from source sensitivity plots calculated with a Lagrangian particle dispersion model and made available online by Stephan Henne at the Swiss Federal Laboratories for Materials Science and Technology (EMPA), Dübendorf, Switzerland (individual results are available on the webpage http://lagrange.empa.ch/FLEXPART_browser/). The surface average temperatures of source areas in the North Atlantic and the Mediterranean Sea were derived from a National Oceanic and Atmospheric Administration NOAA database (http://www.nodc.noaa.gov/OC5/indprod.html), grouped per season, and used to calculate *α*_V/L._

A constant relative humidity factor (*h*) of 0.8 was used for the North Atlantic and for Mediterranean Sea, a value reasonably analogous to those recently reported in Pfahl 2014^36^ since the local meteoric water line shows a deuterium excess comparable to the average precipitations on Earth. Consequently, *α*_V/L_ values were corrected for disequilibrium processes occurring during evaporation from the sea, which tend to increase isotopic fractionation, according to the relationship^37^:


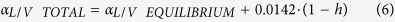


Obtained values for *δ*_V,0_ ranged from −13.76‰ (North Atlantic, winter) to −12.16‰ (Mediterranean Sea, summer), comparable to what is reported in IAEA 2001^18^,^37^.

### Total number of particles N_>0.5_

The total number of particles with a diameter larger 0.5 μm (N_>0.5_) was measured with an optical particle counter (Grimm^TM^, Dust Monitor 1.108). Particles up to 40 μm size are aspired through a heated sample inlet, dried and detected, even when activated as cloud condensation nuclei and part of hydrometeors or ice^38^,^39^. Since the values of N_>0.5_ correspond to unit volume of air, a conversion of INPs per ml of snow into INPs per m^3^ of air was necessary in order to calculate the ratio INPs_−10_/N_>0.5_ presented here. By dividing the precipitation rate measured with the sampling tin by an average deposition velocity of snowflakes (1 m · s^−1^)^40^,^41^, we obtained a value for the snow water equivalent present in 1 m^3^ of air for each sampling interval. A median of 0.25 ml · m^−3^ of snow water equivalent was obtained, well within the range reported by Muhlbauer 2010^42^ and by Deguillaume 2014^43^.

### Statistics

Statistical analyses presented here were done with PAST software version 2.17^44^ and refined with the use of R software version 3.0.1.^45^. Parametric regression was done on logarithmic values of INPs as correction for normality to understand how much of the total variability was covered by our tests and R^2^ values have been reported. These results are accompanied by non-parametric Spearman’s correlation results (r coefficient and p values expressing the probability that variables are not correlated), as a more robust test for the significance of the relationships found. For the comparison among months for the values of INPs a Kruskal-Wallis test was done.

## Additional Information

**How to cite this article**: Stopelli, E. *et al.* Ice nucleation active particles are efficiently removed by precipitating clouds. *Sci. Rep.* **5**, 16433; doi: 10.1038/srep16433 (2015).
